# Understanding Health Risk Comprehension: The Role of Math Anxiety, Subjective Numeracy, and Objective Numeracy

**DOI:** 10.1177/0272989X20904725

**Published:** 2020-02-13

**Authors:** Jonathan J. Rolison, Kinga Morsanyi, Ellen Peters

**Affiliations:** Department of Psychology, University of Essex, Colchester, Essex, UK; School of Psychology, Queen’s University Belfast, Belfast, GB, UK; School of Journalism and Communication, University of Oregon, Eugene, OR, USA

**Keywords:** math anxiety, numeracy, risk comprehension, subjective numeracy

## Abstract

**Background.** Numeracy skills are important for medical decision making as lower numeracy is associated with misinterpreting statistical health risks. Math anxiety, characterized by negative emotions about numerical tasks, and lower subjective numeracy (i.e., self-assessments of numerical competence) are also associated with poor risk comprehension. **Objective.** To explore independent and mediated associations of math anxiety, numerical ability, and subjective numeracy with risk comprehension and to ascertain whether their associations are specific to the health domain. **Methods.** Objective numeracy was measured with a 14-item test. Math anxiety and subjective numeracy were assessed with self-report scales. Risk comprehension was measured with a 12-item test. In experiment 1, risk comprehension items were limited to scenarios in the health domain. In experiment 2, participants were randomly assigned to receive numerically equivalent risk comprehension items in either a health or nonhealth domain. **Results.** Linear regression analyses revealed that individuals with higher objective numeracy were more likely to respond correctly to the risk comprehension items, as were individuals with higher subjective numeracy. Higher math anxiety was associated with a lower likelihood of correct responding when controlling for objective numeracy but not when controlling for subjective numeracy. Mediation analyses indicated that math anxiety may undermine risk comprehension in 3 ways, including through 1) objective numeracy, 2) subjective numeracy, and 3) objective and subjective numeracy in serial, with subjective numeracy mediating the association between objective numeracy and risk comprehension. Findings did not differ by domain. **Conclusions.** Math anxiety, objective numeracy, and subjective numeracy are associated with risk comprehension through unique pathways. Education initiatives for improving health risk comprehension may be most effective if jointly aimed at tackling numerical ability as well as negative emotions and self-evaluations related to numeracy.

People face important decisions about their health care and treatment that often require an understanding of statistical concepts, including percentages, frequencies, and probabilities.^1–4^ Health authorities recommend patient involvement in decisions about their health care and treatment and encourage the provision of statistical information to inform patient decision making.^5,6^ A wealth of research has shown, however, that comprehension of health-related statistical concepts (e.g., lifetime risk, relative risk reduction) is poor among the general public.^1,3,7,8^ Low objective numeracy—assessed with a math test—has been identified as a key factor underlying poor risk comprehension.^3,4,6,7^ Higher math anxiety,^8,9^ which is characterized by negative emotions about performing numerical tasks,^10^ and lower subjective numeracy (self-evaluations of numerical competence) are also associated with poor risk comprehension.^11^ We investigated whether math anxiety, subjective numeracy, and objective numeracy have independent associations with health risk comprehension. Our goal is to shed light on the various pathways to poor risk comprehension to help inform policies aimed at improving patient decision making by targeting the barriers to risk comprehension.

Basic numeracy skills are poor among the general public.^7,12,13,14^ In 1 study, only 57% of a nationally representative sample of adult Americans correctly reported a person’s risk of disease in the next 10 years when the risk was double that of another person, whose risk was 1 in 100.^13^ Individuals with poorer numerical ability are more likely to fail risk comprehension tests, such as by misunderstanding lifetime risks of prostate cancer following genetic testing^3^ or by misinterpreting risk of death from breast cancer with and without mammography.^4^ Subjective numeracy scales, measuring self-reported numerical abilities (e.g., “How good are you at working with percentages?”), have been developed as proxies for objective numeracy, circumventing the need to administer a math test.^11,14^ Fagerlin et al.^11^ proposed that self-assessments of numerical competence could be used to replace objective numeracy measures on the basis of a strong association (*r* = .68) between subjective and objective numeracy measures. However, while objective and subjective numeracy are highly correlated,^11,14^ subjective numeracy scales exhibit low sensitivity and specificity as diagnostic measures of objective numeracy.^15^ As a result, many participants can be identified as either overconfident or underconfident with respect to their numerical abilities.^15^

The findings above suggest that objective and subjective numeracy are independent constructs. Whereas objective numeracy measures ability to perform math tasks, subjective numeracy concerns self-judgments and expectations about one’s ability to perform math tasks. They are linked, of course. Successful performance on a task demonstrates skills and abilities to perform similar tasks in the future, which, in turn, increases self-efficacy (self-assessments of one’s ability to perform similar tasks).^16,17^ Self-efficacy is a strong predictor of task performance, in part owing to effects of self-efficacy on investment of effort and persistence with challenging tasks.^16,18^ Therefore, higher objective numerical ability may increase subjective numerical ability and, in turn, improve performance on risk comprehension tasks through greater effort and persistence. Indeed, the association between objective numeracy and decision outcomes has been shown to be mediated by subjective numeracy.^19,20^ Therefore, we hypothesized that direct associations of each numeracy would exist with risk comprehension and that higher subjective numeracy would partially mediate the association between objective numeracy and risk comprehension.

Math anxiety refers to feelings of tension, fear, or apprehension that affect performance on math tasks.^10^ It is associated with poorer comprehension of statistical health risks.^8,9^ Individuals who are higher in math anxiety typically attain lower scores on tests of numerical ability,^21^ which may be due partially to avoidance of opportunities for math education.^22^ Anxiety experienced during engagement with math tasks may also interfere with performance by distracting or occupying limited working memory resources that are necessary for good performance.^23–25^ Rolison et al.^8^ found that higher math anxiety was associated with poorer interpretation of absolute and relative risk reductions but not after controlling for objective numeracy, indicating that objective numeracy mediated an association between math anxiety and risk comprehension. Other studies have found evidence of objective numeracy partially mediating the association between math anxiety and performance with numerical reasoning tasks (e.g., the cognitive reflection test), with a significant direct link between math anxiety and performance.^26,27^ This finding suggests a possible direct association between math anxiety and performance independent of numerical ability.

A relationship also exists between math anxiety and other forms of anxiety, including test anxiety and generalized anxiety.^22,28^ Nevertheless, math anxiety remains correlated with math performance after controlling for test anxiety and generalized anxiety,^22^ confirming its distinct association with math performance. Health anxiety, which is characterized by unrealistic concerns about one’s health, is correlated with various other anxiety disorders.^29^ In the Rolison et al.^8^ study, the association between math anxiety and comprehension of statistical health risks may have been confounded by comorbid anxieties—namely, health anxiety—provoked by the narrative content of the health risk comprehension problems. We investigated whether math anxiety is associated with risk comprehension even after controlling for health anxiety and generalized anxiety.

Less well known is the relation of math anxiety with subjective numeracy. However, in Rolison et al.,^8^ math anxiety was more strongly associated with confidence in comprehension than with correct comprehension, such that math-anxious individuals were less confident in their comprehension. Investigations of math anxiety in educational contexts have also found strong correlations between measures of math anxiety and confidence.^22^ As confidence in one’s performance is closely related to self-assessments of one’s ability to perform a task, subjective numeracy may mediate the association between math anxiety and risk comprehension. That is, anxiety, tension, and fear associated with math anxiety may have detrimental effects on self-evaluations of math ability, reducing subjective numeracy and, in turn, worsening persistence on numeric tasks and risk comprehension. We hypothesized a direct association between math anxiety and subjective numeracy on risk comprehension and a mediating role of subjective numeracy on the association between math anxiety and risk comprehension in experiments 1 and 2.

Finally, we question whether the pathways to poor risk comprehension are specific to the health domain. Some theorists have proposed that health numeracy is a separate competency to general numerical ability.^30–32^ Levy et al.,^32^ for example, found that participants were less likely to respond correctly to math problems presented in the health domain (e.g., percentage of people who get a disease) compared to a financial (e.g., percentage of customers who get a discount) or pure math (i.e., no risk context) domain. One possible explanation for this finding is that due to its importance, health-related information provokes anxiety that interferes with risk comprehension. Adverse effects of health-related content on risk comprehension should be stronger among health-anxious individuals who are likely to be more sensitive to health-related information and among individuals who are high in math anxiety as any anxiety provoked by the verbal content of a problem would exacerbate anxiety caused by its numerical content. Therefore, in experiment 2, we further explored whether associations between math anxiety, subjective numeracy, and objective numeracy differ depending on the domain of risk comprehension problems.

In sum, the current investigation was designed to test for independent associations between math anxiety, subjective numeracy, and objective numeracy with risk comprehension. We hypothesized that the association between math anxiety and risk comprehension would be mediated by 1) objective numeracy, 2) subjective numeracy, and 3) objective and subjective numeracy in serial, whereby subjective numeracy mediates the association between objective numeracy and risk comprehension. In addition, we explored whether the associations between objective numeracy, math anxiety, and subjective numeracy depend on the domain of risk comprehension problems.

## Experiment 1

### Method

#### Participants

In total, 1257 participants were invited to participate in a study of their understanding of statistical health risks using online public and private recruitment platforms. Of these, 1194 consented to participate and 1011 participants competed the study. Only complete data were used in all analyses. Of those who completed the study, 660 were recruited via Amazon’s Mechanical Turk, and the remaining 351 were recruited either on a voluntary basis or in exchange for course credit. Most (*n* = 705) were from the United States or Canada, 244 were from the United Kingdom or Ireland, and a minority (*n* = 59) were from another country. Table 1 provides the sample characteristics.

**Table 1:** Participant Demographics

Characteristic	Experiment 1 (*n* = 1011)	Experiment 2 (*n* = 940)
Age, mean (SD), y	33.77 (11.77)	30.42 (11.76)
Age range, y	18–74	18–70
Female sex, %	61	71
Highest educational attainment, %		
High school	11	12
Some college	41	41
University degree	31	33
Postgraduate course	18	12
Employment, %		
Full-time	50	38
Part-time	21	25
Unemployed	10	17
Other occupation (e.g., homemaker)	20	20
Place of birth, %		
United States or Canada	70	49
United Kingdom or Republic of Ireland	24	26
Other	6	25

#### Materials and procedure

##### Objective numeracy

Objective numeracy was assessed with the 11-item Lipkus et al.^7^ scale and 3 cognitive reflection items (see Appendix A).^33^ The Lipkus et al.^7^ scale includes 3 items that assess general understanding of chance and probability and 8 items that assess understanding of disease risk, such as converting percentages to frequencies.^7^ The cognitive reflection items assess the ability to produce a numerically correct response by applying a normative rule and resisting an intuitively appealing response.^33^ We combined the Lipkus et al.^7^ scale items and cognitive reflection items to extend the scale’s range of difficulty as total scores tend to be negatively skewed toward the high end of the scale for the Lipkus et al.^7^ scale items.^13,34^ Confirmatory and exploratory factor analysis have shown that cognitive reflection items are appropriate to use with standard numeracy questions as they load on the same numerical ability factor as the Lipkus et al.^7^ scale items.^35–38^ Previous studies have included Cognitive Reflection Test (CRT) items with the Lipkus et al.^7^ scale items due to improvements in the scale structure and reliability.^36,37^ Items were scored as either correct (value of 1) or incorrect (value of 0). Total scores were summed across the 11 Lipkus et al.^7^ scale items and the 3 cognitive reflection items (Cronbach α = .80).

##### Subjective numeracy

Subjective numeracy was assessed with an 8-item scale developed by Fagerlin et al.^11^ The scale assesses self-reported ability to work with numerical information (e.g., “How good are you at working with percentages?”) on a 6-point scale, ranging from *not at all good* (value of 1) to *extremely good* (value of 6), and preferences for numerical formats of information (e.g., “How often do you find numerical information to be useful? [1 = *never*, 6 = *very often*]) on a 6-point scale. Overall subjective numeracy was calculated as the mean score across the 8 items (Cronbach α = .87).

##### Math anxiety

Math anxiety was assessed with the 13-item Adult Everyday Math Anxiety Scale (AEMAS),^8^ which evaluates self-reported anxiety with numerical information in general (e.g., “having to work with percentages”), in everyday tasks (e.g., “having to work out prices in a foreign currency”), and in the workplace (e.g., “having to present numerical information at a work meeting”). Participants responded on a 5-point scale, ranging from *low anxiety* (value of 1) to *high anxiety* (value of 5). Overall math anxiety was calculated as the mean score across the 13 items (Cronbach α = .93).

##### Generalized anxiety

Generalized anxiety was assessed with the 7-item Generalized Anxiety Disorder Scale,^39^ which assesses mild to severe levels of generalized anxiety based on self-reported frequency of anxiety symptoms over the last 2 weeks (e.g., “feeling nervous, anxious, or on the edge”) on a 3-point scale, ranging *not at all* (value of 1) to *nearly every day* (value of 4). Overall generalized anxiety was calculated as the mean score across the 7 items (Cronbach α = .92).

##### Health anxiety

Health anxiety was assessed with the 15-item Health Anxiety Questionnaire,^40^ which measures health concerns, preoccupation with health issues, attention to aches and pains and bodily sensations, and fear of serious illness on a 4-point scale (e.g., *not at all or rarely* [value of 1], *sometimes* [value of 2], *often* [value of 3], *most of the time* [value of 4]). Overall health anxiety was calculated as the mean score across the 15 items (Cronbach α = .93).

##### Risk comprehension

We constructed a battery of 12 risk comprehension items in the health domain based on novel items and items drawn from the existing literature (see Appendix A for full list of items). Items assessed comprehension of absolute risk (“the patient’s chance of surviving . . . is increased to 70%”; question 1),^8^ relative risk (“the patient’s chance of surviving . . . is increased by 25%”; question 2),^8^ and lifetime risk of cancer informed by genetic testing (question 3).^3^ Novel items assessed comprehension of ratios in the context of communicating the health benefits of a vitamin supplement (question 4), misconceptions relating to random event sequences in the context of the most likely outcome for a patient in a hospital who follows a sequence of prior patients (question 5), and proportions in terms of the percentage of people who are at increased risk of developing a serious health condition (question 6). We also included items that assessed comprehension of comparative information in the context of multiple performance indicators of hospitals (questions 7–12).^41^

For example, the item that assessed comprehension of event sequences (question 5) asked participants the following:

In a hospital, 10 in every 30 patients who undergo a medical procedure require further treatment and the remaining 20 do not require any further treatment. The last 5 medical procedures carried out in the hospital did not require any further treatment. What do you think is the most likely outcome for the next patient who undergoes a medical procedure in the hospital?

Option 1: The patient will not require further treatment.
Option 2: The patient will require further treatment.
Option 3: The patient has equal chances that they will or will not require further treatment.

The risk comprehension items were scored as either correct (value of 1) or incorrect (value of 0). Total scores were summed across all 12 items (Cronbach α = .69).

Participants first completed the generalized anxiety scale. They then completed the health anxiety scale, followed by the subjective numeracy scale, then the math anxiety scale, followed by the risk comprehension items, and, finally, the objective numeracy scale. The risk comprehension and objective numeracy items were presented after the math anxiety scale and subjective numeracy scale to avoid influencing participants’ self-reported math anxiety and subjective numeracy.

### Results

Participants responded correctly to a mean (SD) of 8.61 (2.20) of the 12 risk comprehension items. Table 2 provides the intercorrelations among variables. Higher risk comprehension scores were associated with higher objective and subjective numeracy and lower math anxiety, health anxiety, and generalized anxiety. Higher objective numeracy was associated with higher subjective numeracy and lower math anxiety, health anxiety, and generalized anxiety. Math anxiety was positively associated with health anxiety and generalized anxiety.

**Table 2:** Experiment 1: Descriptive Statistics and Pearson Correlations (*n* = 1194)^a^

Characteristic	Mean (SD)	(1)	(2)	(3)	(4)	(5)	(6)	(7)	(8)	(9)
Age (1)	33.76 (11.77)	—								
Male sex (2)	*n* = 398 (39%)	−.01	—							
Education (3)	1.56 (0.90)	.04	−.10^b^	—						
Objective numeracy (4)	10.54 (2.83)	−.01	−.12^c^	.25^c^	(.80)					
Subjective numeracy (5)	4.40 (1.01)	.07^b^	.24^c^	.11^c^	.52^c^	(.87)				
Math anxiety (6)	1.97 (0.79)	−.09^b^	−.20^c^	−.15^c^	−.44^c^	−.62^c^	(.93)			
Health anxiety (7)	1.72 (0.54)	−.10^c^	−.05	−.13^c^	−.23^c^	−.19^c^	.43^c^	(.93)		
Generalized anxiety (8)	1.83 (0.75)	−.22^c^	−.14^c^	−.07^b^	−.16^c^	−.21^c^	.40^c^	.52^c^	(.92)	
Risk comprehension (9)	8.60 (5.05)	.02	.01	.23^c^	.70^c^	.45^c^	−.35^c^	−.17^c^	−.09^b^	(.69)

a sCronbach α values are shown in parentheses. Education was coded as 0, high school; 1, some college; 2, university degree; 3, postgraduate degree.

b *P*≤ .05, 2-tailed significance.

c *P*≤ .001, 2-tailed significance.

#### Multiple linear regression analysis on risk comprehension

Provided in Table 3 are the results of our linear regression analysis on total risk comprehension scores. Age, sex, education, objective numeracy, math anxiety, health anxiety, and generalized anxiety were included in model 1a. Subjective numeracy was included in model 2a to assess effects of math anxiety after controlling for subjective numeracy. Higher objective numeracy was associated with higher risk comprehension scores (model 1a; Table 3). Controlling for objective numeracy, higher math anxiety was associated with lower risk comprehension scores (model 1a; Table 3). Controlling for health anxiety and generalized anxiety, math anxiety remained a significant predictor, while health anxiety and generalized anxiety were not (model 1a; Table 3). Higher subjective numeracy was associated with higher risk comprehension scores when included in a second model (model 2a; Table 3). Controlling for subjective numeracy, math anxiety was no longer significantly associated with risk comprehension (model 2a; Table 3). (The pattern of results was similar when the objective numeracy measure included only the 11 Lipkus et al.^7^ scale items, with the exception that education was positively associated with risk comprehension in model 1a [*b* = .15, *t* = 2.52, *P* = 0.012] and model 2a [*b* = .16, *t* = 2.70, *P* = 0.007].) In sum, as hypothesized, objective and subjective numeracy each had direct associations with risk comprehension. Math anxiety was associated with risk comprehension independent of objective numeracy, health anxiety, and generalized anxiety, but its association with risk comprehension appeared to be mediated by subjective numeracy. Health anxiety and generalized anxiety were not associated with risk comprehension independent of math anxiety.

**Table 3:** Linear Regression Models Used to Predict Risk Comprehension Scores^a^

Included	Experiment 1 (*n* = 1194), Unstandardized β		Included	Experiment 2 (*n* = 940), Unstandardized β		
	Model 1a	Model 2a		Model 1b	Model 2b	Model 3b
Age	0.01	0.00	Age	−0.01	−0.01	−0.01
Male sex	−0.35^b^	−0.42^c^	Male sex	−0.12	−0.17	−0.17
Objective numeracy	0.52^c^	0.48^c^	Objective numeracy	0.45^c^	0.43^c^	0.47^c^
Education	0.10	0.11	Education	0.12	0.10	0.10
Math anxiety	−0.22^b^	0.00	Math anxiety	−0.27^c^	−0.14	−0.15
Health anxiety	−0.01	−0.07	Health anxiety	−0.15	−0.21	−0.23
Generalized anxiety	0.12	0.12	Domain	−0.01	0.00	0.22
Subjective numeracy		0.33^c^	Subjective numeracy		0.21^c^	0.17
			Objective Numeracy × Domain			−0.07
			Math Anxiety × Domain			0.02
			Health Anxiety × Domain			0.03
			Subjective Numeracy × Domain			0.08

a Education was coded as 0, high school; 1, some college; 2, university degree; 3, postgraduate degree.

b *P*≤ .05.

c *P*≤ .001.

#### Mediation analysis on risk comprehension

We hypothesized that the association between math anxiety and risk comprehension would be mediated by 1) objective numeracy, 2) objective and subjective numeracy in serial, and 3) subjective numeracy. To test our mediation hypotheses, we employed Preacher and Hayes’s INDIRECT regression procedure with 10,000 bootstrapped samples to estimate the 95% confidence intervals (CIs) for the direct and indirect pathways.^42^ (This procedure makes it possible to test the potential effects of a number of mediators, as well as potential serial mediation effects, in a single analysis, without the need to conduct separate analyses to statistically compare the adequacy of competing models.)

**Figure 1:**
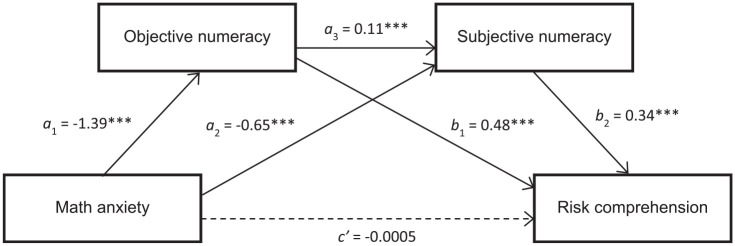
Mediation analysis. The model assessed effects of math anxiety on risk comprehension via objective numeracy (*a*_1_*b_1_ = indirect pathway 1), subjective numeracy (*a*_2_*b_2_ = indirect pathway 3), and objective and subjective numeracy (*a*_1_*a_3_*b_2_ = indirect pathway 2), as well as the unmediated direct effect (*c*′) of math anxiety on risk comprehension. Sex, education, generalized anxiety, and health anxiety were included as covariates.

In our mediation model (Figure 1), we estimated the indirect pathway between math anxiety and risk comprehension via objective numeracy (indirect pathway 1), objective and subjective numeracy in serial (indirect pathway 2), and via subjective numeracy (indirect pathway 3). In our analysis, we controlled for health anxiety and generalized anxiety to confirm the specific associations of math anxiety (as opposed to a more general anxious predisposition) with risk comprehension. We controlled for sex, as math anxiety is often more prevalent in women, whereas men are often characterized by higher levels of subjective numeracy, which was also the case in the current sample (Table 2). We also controlled for education as higher education was associated with lower math anxiety and higher objective and subjective numeracy (Table 2). In the INDIRECT regression procedure, a bias-corrected bootstrapped CI of the product of the paths within each indirect route that does not include zero indicates a significant indirect association of math anxiety with risk comprehension through the mediating variables.^42^

The total effect of math anxiety on risk comprehension was significant (*c* = −.939; 95% CI, −1.122 to −0.756; *P* < 0.001). Nevertheless, once the mediators were entered into the regression, the direct association between math anxiety and risk comprehension was no longer significant (*P* = 0.995). In addition, our mediation analysis revealed that all 3 indirect pathways were significant. Specifically, there was a significant indirect association of math anxiety with risk comprehension via objective numeracy (i.e., indirect pathway 1; *b* = −0.669; 95% CI, −0.816 to −0.537), objective and subjective numeracy in serial (indirect pathway 2; *b* = −0.521; 95% CI, −0.081 to −0.031), and via subjective numeracy (indirect pathway 3; *b* = −0.217; 95% CI, −0.313 to −0.129; Figure 1). The ratio of the indirect to the total effect can be used as an effect size statistic for the mediation effects.^43^ These results indicated that the mediational effect of objective numeracy for math anxiety was large (.71), whereas the mediational effects of objective and subjective numeracy in serial (.06) and subjective numeracy (.23) were small. Regarding the covariates, education was a significant covariate (*P* < 0.0001; 95% CI, 0.267−0.549); the effect of generalized anxiety (*P* = 0.055; 95% CI, −0.005 to 0.396) approached significance, whereas sex (*P* = 0.212) and health anxiety (*P* = 0.230) were nonsignificant covariates. (The pattern of results was the same when the objective numeracy measure included only the 11 Lipkus et al.^7^ scale items.) In sum, our mediation analysis supported our mediation hypotheses, demonstrating that objective and subjective numeracy mediated the association between math anxiety and risk comprehension and that subjective numeracy partially mediated the association between objective numeracy and risk comprehension.

## Experiment 2

In experiment 2, we aimed to replicate the findings of experiment 1, indicating that objective and subjective numeracy mediate an association between math anxiety on risk comprehension in the health domain. Previous research has indicated that people perform more poorly on math problems when presented in the health domain compared to other domains.^32^ A further aim of experiment 2 was to explore whether the associations between objective numeracy, math anxiety, subjective numeracy, and risk comprehension differ depending on the domain of risk comprehension problems. In experiment 2, participants were randomly assigned to receive risk comprehension problems with identical numerical content in either the health domain, as in experiment 1, or in a nonhealth domain.

### Method

#### Participants

In total, 1423 participants were invited to participate in a study of their understanding of statistical health risks using online public and private recruitment platforms. Of these, 1261 consented to participate and 940 participants competed the study. Only complete data were used in all analyses. Of those who completed the study, 225 were recruited via Amazon’s Mechanical Turk, and the remaining 715 were recruited either on a voluntary basis or in exchange for course credit. Most (*n* = 463) were from the United States or Canada, 244 were from the United Kingdom or Ireland, and the remaining 233 were from another country. Table 1 provides the sample characteristics.

#### Materials and procedure

As in experiment 1, participants completed the objective numeracy, subjective numeracy, math anxiety, and health anxiety scales. Experiment 1 demonstrated that math anxiety was a significant predictor of risk comprehension after controlling for the effects of generalized anxiety and health anxiety. Nevertheless, we included the health anxiety scale as a covariate in experiment 2, as we were interested in potential differences in the effects of math anxiety on risk comprehension between the health and nonhealth domains after controlling for potential effects of health anxiety.

##### Risk comprehension

We constructed an alternative format of the 12 health-related risk comprehension items used in experiment 1. In our alternative format, the scenarios were altered such that they no longer referred to health. For example, rather than refer to a patient’s chance of survival 1 year after a cancer diagnosis (health domain), the equivalent scenario in the nonhealth domain referred to a toy shop’s chance of making a profit 1 year after the sale of a new product (see Appendix A). Importantly, the nonhealth version of each item maintained an identical structure, had a similar length, and presented identical numerical information. Hence, the items in the health domain and nonhealth domain were identical in all aspects other than their reference to health- or non-health-related scenarios.

Participants first completed the health anxiety scale, followed by the subjective numeracy scale, math anxiety scale, risk comprehension items, and, finally, the objective numeracy scale. Participants were randomly assigned to complete either the health (*n* = 476; 50%) or nonhealth version of the risk comprehension items.

### Results

Participants responded correctly to a similar number of risk comprehension items in the health ($\bar{x}$ = 8.16, *s* = 2.18) and nonhealth ($\bar{x}$ = 7.98, *s* = 2.25) domains (*P* = 0.203). Across domains, higher risk comprehension scores were associated with higher objective and subjective numeracy and lower math anxiety and health anxiety (Table 4). Higher objective numeracy was associated with higher subjective numeracy and lower math and health anxiety. Math anxiety was positively associated with health anxiety (Table 4). Thus, the intercorrelations among the variables replicated the findings of experiment 1.

**Table 4:** Experiment 2: Descriptive Statistics and Pearson Correlations (*n* = 940).^a^

Characteristic	Mean (SD)	(1)	(2)	(3)	(4)	(5)	(6)	(7)	(8)
Age (1)	30.42 (11.76)	—							
Male sex (2)	*n* = 277 (29%)	.05	—						
Education (3)	1.46 (0.86)	.13^b^	−.07^c^	—					
Objective numeracy (4)	9.33 (2.85)	−.02	.05	.07^c^	(.75)				
Subjective numeracy (5)	4.11 (1.07)	.06	.21^b^	.14^b^	.42^b^	(.84)			
Math anxiety (6)	2.16 (0.86)	−.09^c^	−.19^b^	−.10^c^	−.44^b^	−.54^b^	(.93)		
Health anxiety (7)	1.84 (0.56)	−.12^b^	−.04	−.05	−.26^b^	−.11^b^	.43^b^	(.93)	
Risk comprehension (8)	8.07 (2.12)	−.04	.02	.10^c^	.65^b^	.37^b^	−.38^b^	−.21^b^	(.67)

Cronbach α values are shown in parentheses. Education was coded as 0, high school; 1, some college; 2, university degree; 3, postgraduate degree.

b *P*≤ .001, 2-tailed significance.

c *P*≤ .05, 2-tailed significance.

#### Multiple linear regression analysis on risk comprehension

Provided in Table 3 are the results of our linear regression analysis on total risk comprehension scores. Age, sex, education, objective numeracy, math anxiety, health anxiety, and domain were included in model 1b. Subjective numeracy was included in model 2b to assess effects of math anxiety after controlling for subjective numeracy. Interaction terms involving domain were included in model 3b to test for moderating effects of domain on objective numeracy, math anxiety, health anxiety, and subjective numeracy. Higher objective numeracy was associated with higher risk comprehension scores (model 1b; Table 3). Controlling for objective numeracy, higher math anxiety was associated with lower risk comprehension scores (model 1b; Table 3). Controlling for math anxiety, health anxiety was not significantly associated with risk comprehension (model 1b; Table 3). Moreover, risk comprehension did not differ depending on whether the scenarios related to the health or nonhealth domain (model 1b; Table 3). In a second model, higher subjective numeracy was associated with higher risk comprehension scores, and controlling for subjective numeracy, math anxiety was no longer significantly associated with risk comprehension (model 2b; Table 3). In our final model (model 3b; Table 3), domain (i.e., health v. nonhealth) did not moderate effects of objective numeracy, math anxiety, health anxiety, or subjective numeracy on risk comprehension scores. (The pattern of results was similar when the objective numeracy measure included only the 11 Lipkus et al.^7^ scale items, with the exceptions that education was positively associated with risk comprehension [*b* = .14, *t* = 2.13, *P* = 0.034] in model 1b and that math anxiety [*b* = −.21, *t* = 2.31, *P* = 0.021] and health anxiety [*b* = −.23, *t* = 2.00, *P* = 0.045] were associated with poorer risk comprehension in model 2b.) In sum, our multiple linear regression analysis replicated experiment 1’s findings and revealed no effects of risk comprehension domain.

#### Mediation analysis on risk comprehension

**Figure 2:**
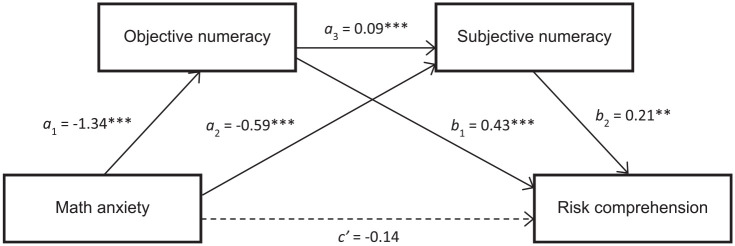
Mediation analysis. The model assessed effects of math anxiety on risk comprehension via objective numeracy (*a*_1_*b_1_ = indirect pathway 1), subjective numeracy (*a*_2_*b_2_ = indirect pathway 3), and objective and subjective numeracy (*a*_1_*a_3_*b_2_ = indirect pathway 2), as well as the unmediated direct effect (*c*′) of math anxiety on risk comprehension. Sex, education, and health anxiety were included as covariates. Generalized anxiety was removed from the model vis-à-vis Figure 1.

In our mediation model (Figure 2), we followed the procedure introduced in experiment 1 to test the indirect effect of math anxiety on risk comprehension via objective numeracy (indirect pathway 1), objective and subjective numeracy in serial (indirect pathway 2), and via subjective numeracy (indirect pathway 3). Sex, education, and health anxiety were included as covariates. The total effect of math anxiety on risk comprehension was significant (*c* = −.868; 95% CI, −1.039 to −0.696; *P* < 0.0001). Nevertheless, once the mediators were entered into the regression, the direct effect of math anxiety was no longer significant (*P* = 0.108). Our mediation analysis confirmed that all 3 indirect effects were significant. Specifically, there was a significant indirect effect of math anxiety on risk comprehension via objective numeracy (i.e., indirect pathway 1; *b* = −0.581; 95% CI, −0.702 to −0.473), objective and subjective numeracy in serial (indirect pathway 2; *b* = −0.025; 95% CI, −0.045 to −0.010), and via subjective numeracy (indirect pathway 3; *b* = −0.122; 95% CI, −0.208 to −0.049). (We also tested for moderating effects of domain [health v. nonhealth] on the indirect pathways, which yielded no moderating effects.) The ratios of the indirect to the total effect indicated that the mediational effect of objective numeracy for math anxiety was large (.67), whereas the mediational effects of objective and subjective numeracy in serial (.03) and subjective numeracy (.14) were small. Regarding the covariates, health anxiety was the only significant covariate (*P* = 0.027; 95% CI, −0.551 to −.033), whereas the effect of education approached significance (*P* = 0.078). (The pattern of results was similar when the objective numeracy measure included only the 11 Lipkus et al.^7^ scale items, with the exception that the direct effect of math anxiety on risk comprehension remained significant after including the mediators and covariates in the model [*b* = −0.201; 95% CI, −0.375 to −0.027]. That is, when only the easier numeracy items were included in the numeracy scale, the effect of math anxiety was only partially mediated.) In sum, our mediation analysis replicated experiment 1’s findings regarding the indirect effects of math anxiety and objective numeracy on risk comprehension.

## General Discussion

What are the barriers to comprehension of statistical health risks? Previous research has identified objective numeracy,^4^ subjective numeracy,^11^ and math anxiety^8^ as predictors of risk comprehension. Yet, no previous study has explored whether these constructs have independent associations with risk comprehension. In the current investigation, we explored the effects of math anxiety, subjective numeracy, and objective numeracy together to shed light on the determinants of poor risk comprehension. We found that subjective and objective numeracy were directly associated with risk comprehension. Math anxiety was directly associated with risk comprehension when controlling for objective numeracy but not when controlling for both objective and subjective numeracy. We discovered 3 indirect pathways of math anxiety to risk comprehension, including via objective numeracy, subjective numeracy, and via objective and subjective numeracy in serial, whereby subjective numeracy mediated effects of objective numeracy after controlling for effects of math anxiety on objective numeracy.

Rolison et al.^8^ reported that higher math anxiety was associated with poorer comprehension of absolute and relative risk reductions associated with medical treatments but not after controlling for objective numeracy. Our current findings replicate the previously reported mediating effect of objective numeracy, even after controlling for individual differences in health and generalized anxiety. This finding implies that the effect of anxiety on objective numeracy is specific to anxiety about math problems. The indirect effect of math anxiety is likely to be a consequence of the tendency for math-anxious individuals to rate their skills as lower, have less confidence, and avoid opportunities to respond to current math-related problems or to take advantage of earlier math education, limiting their development of numeracy skills.^21,22^

We also found an effect of math anxiety on risk comprehension after controlling for objective numeracy and health and generalized anxiety. We speculate that the effect of math anxiety on risk comprehension after controlling for objective numeracy may not have been detected in the Rolison et al.^8^ study because the present study used a much larger battery of risk comprehension items, increasing statistical power and reducing the extent to which our findings depend on a single risk comprehension problem. Moreover, our findings show that effects of math anxiety remain even after controlling for health and generalized anxiety, indicating that anxiety is specific to the numerical content of risk comprehension problems.

The effects of math anxiety on risk comprehension, however, were mediated by subjective numeracy. This novel finding suggests a pathway to misinterpretation of statistical health risks that is independent of numeracy skills or abilities. We speculate that anxiety about numerical content negatively affects self-evaluations of math ability (i.e., subjective numeracy), which, in turn, worsens performance on risk comprehension tasks through reduced effort or persistence. Education initiatives targeted at improving numeracy skills may be undermined if they fail also to address people’s anxieties about math and negative self-evaluations. Hence, an important implication of our findings is that education programs may be most effective if they stretch beyond training basic numeracy skills and address emotions and self-evaluations of abilities. Successful performance on a task improves self-evaluations of one’s ability to perform related tasks.^16^ University undergraduates who received an intervention designed to increase math-related self-efficacy, which included basic numerical problem-solving tasks, subsequently reported greater confidence in their ability to perform math-related tasks and expressed greater interest in studying math- or science-related courses.^44^ Moreover, among young children, modifying math problems to enable high student success rates increases subsequent math performance by motivating more practice.^45^ One initiative could involve using similar techniques in high school and university-level math education to improve self-evaluations and alleviate math anxiety through performance accomplishment. Such efforts may be particularly important to health when good outcomes depend on numeric ability but also persistence over time.^46^

The current findings imply a multifaceted nature of numerical competencies underlying risk comprehension. Subjective numeracy scales have often been used as a proxy for actual numerical abilities,^11,14^ despite offering a poor diagnostic tool for assessing objective numeracy.^15^ In the current experiments, objective numeracy had a direct effect on risk comprehension and an indirect effect via subjective numeracy, implying that objective and subjective numeracy have independent associations with risk comprehension even though they are related. The serial pathway from objective numeracy to risk comprehension via subjective numeracy has been supported in other studies by structural equation model analysis in which reversing the path between objective numeracy and subjective numeracy results in a poorer model fit.^19^ Similarly, in an intervention study designed to improve numeracy with a statistics course combined with values affirmation, the alternative model with a pathway leading from subjective numeracy to risk comprehension via objective numeracy fitted the data less well than a pathway leading from objective numeracy to risk comprehension via subjective numeracy.^20^ A clinical implication of our findings is that subjective numeracy may be an inadequate proxy for numerical ability as it does not fully account for the association between objective numeracy and risk comprehension. The direct effect of subjective numeracy on risk comprehension (even after controlling for effects of math anxiety and numeracy) also has potential clinical importance. Higher self-efficacy (i.e., self-judgments of ability) leads to better task performance as a consequence of greater persistence and investment of effort.^16,18^ If subjective numeracy levels were enhanced with an intervention designed to reduce negative self-evaluations, this could lead to better risk comprehension, improving patient decision making in health contexts. Care needs to be taken, however, as such efforts could increase overconfidence. A fruitful avenue for future research would be to explore how interventions designed to enhance subjective numeracy affect performance on risk comprehension tasks.

Levy et al.^32^ reported that performance on math problems posed in the health domain was poorer than for problems that had a financial or purer math content. Their finding resonates with a view that health numeracy is a separate construct to general numerical ability.^30–32^ A possible interpretation of their finding is that health-related information provokes anxiety that interferes with performance. However, using a larger battery of risk comprehension problems (i.e., 12 items) than Levy et al.^32^ (4 items), we did not find differences in risk comprehension between problems posed in health and nonhealth domains. Moreover, effects of math anxiety, subjective numeracy, and objective numeracy did not depend on domain, suggesting that they each have domain-general effects on risk comprehension. As discussed below, participants in the current experiments reported relatively low symptoms of health anxiety. If future research were to assess individuals of higher health anxiety (e.g., with an illness anxiety disorder), domain differences in health comprehension may occur due to impairing effects of anxiety.

The current research has potential limitations. Our mediation analysis was correlational in nature, which precludes strong claims about the directionality of some pathways within our mediation model. As discussed earlier, the serial pathway from objective numeracy to risk comprehension via subjective numeracy has been supported by previous research.^19,20^ Thus, we took a confirmatory approach to test this pathway in our experiments. However, our approach does not rule out alternative models, such as a pathway leading from subjective numeracy to risk comprehension via objective numeracy, which would imply that negative self-assessments of math ability undermine performance on math problems, leading to poor risk comprehension. Further research could seek to manipulate subjective numeracy (e.g., by presenting easy or difficult math problems) to unpick its causal links with objective numeracy, math anxiety, and risk comprehension. We focused our investigation on individuals in the general population. On average, participants reported experiencing relatively low symptoms of health anxiety in experiment 1 ($\bar{x}$ = 1.72; Table 2) and experiment 2 ($\bar{x}$ = 1.84; Table 4), where 1 = *not at all or rarely* and 2 = *sometimes*. However, patients with a health-related anxiety disorder (e.g., illness anxiety disorder) exhibit considerably higher health anxiety scores than the general public.^47,48^ High levels of health anxiety, as exhibited by patients who have illness anxiety disorder, may have negative effects on comprehension of statistical health risks missed by the relatively low levels of health anxiety we observed presently. A valuable direction for future research would be to explore whether anxiety experienced by illness anxiety disorder patients influences health risk comprehension independent of the effects of math anxiety. Patients who score high in health anxiety visit their physician more frequently than other patients,^49,50^ and people with illness anxiety disorder search more online for health-related information.^50^ Thus, individuals with this disorder are much more exposed to health statistics than others, and their potentially poor comprehension of such information may exacerbate their health anxieties.

A third of participants had completed a university degree. In both experiments, higher educational attainment was associated with lower math anxiety, higher subjective and objective numeracy, and better risk comprehension. Thus, the high educational attainment of our samples may have suppressed an even stronger association between math anxiety, subjective and objective numeracy, and risk comprehension. Future research could target individuals with low educational attainment where math anxiety is likely to be higher and subjective and objective numeracy lower, addressing a sample of the population who is likely to misunderstand numerical health risks. The percentage of participants who failed to complete experiments 1 and 2 (15% and 25%, respectively) was considerable, and thus, effort should be made to maximize participant completion rates if specialist samples are sought in future research.

Finally, we measured objective numeracy with the 11-item Lipkus et al.^7^ scale and 3 additional CRT items in a manner similar to a well-validated Rasch-based measure.^37^ The Lipkus et al.^7^ scale is perhaps the most widely used scale to assess objective numeracy in the context of health risk comprehension, and scores on the scale have been shown to correlate highly with subjective numeracy,^11,14^ math anxiety,^8^ and risk comprehension.^3,4^ However, studies have reported that scores on the scale are negatively skewed toward the high end of the scale.^13,34^ We included 3 additional CRT items in our objective numeracy measure, on which performance is typically poorer,^33^ to address the scale’s skewed scores and to capture a broader range of numerical ability. While alternative measures exist, such as the Berlin Numeracy Test,^51^ designed to overcome the psychometric problems with the Lipkus et al.^7^ scale, studies nevertheless have shown stronger positive associations between subjective numeracy and objective numeracy measured using the Lipkus et al.^7^ scale than the Berlin Numeracy Test.^52^ Some researchers have questioned the inclusion of CRT items with items of numeracy scales.^53^ However, previous studies have shown that CRT items load on the same factor as the Lipkus et al.^7^ scale items and improve scale structure and reliability when combined.^35–38^ Moreover, our pattern of results for both experiments was similar when we excluded the CRT items and our objective numeracy scale included only the Lipkus et al.^7^ scale items. The Lipkus et al.^7^ scale comprises a mixture of health- and non-health-related items. A previous study reported poorer performance on math problems presented in the health domain compared to other domains.^32^ Using a larger battery of items, in experiment 2, we did not find any differences in risk comprehension for health- and non-health-related items, and the associations between math anxiety, objective numeracy, subjective numeracy, and risk comprehension did not differ with domain. Thus, it is unlikely that our findings, or those of other studies, were affected by the Lipkus et al.^7^ scale containing a mixture of health- and non-health-related items.

In conclusion, the current findings suggest that math anxiety, objective numeracy, and subjective numeracy are independent constructs that each relate to comprehension of statistical health risks via unique pathways. These findings indicate a multifaceted nature of numerical competencies in the health context and highlight a need to move beyond singular predictors (e.g., objective numeracy) to investigate indirect pathways to risk comprehension. We discovered pathways to poor risk comprehension that were independent of numeracy skills. This finding implies that government policies and education initiatives may be most effective if targeted at math emotions and self-evaluations, in addition to training math skills, recognizing the multifaceted nature of numerical competence.

## Supplemental Material

RiskCompAppendix2019.rjf_online_supp – Supplemental material for Understanding Health Risk Comprehension: The Role of Math Anxiety, Subjective Numeracy, and Objective Numeracy Click here for additional data file. Supplemental material, RiskCompAppendix2019.rjf_online_supp for Understanding Health Risk Comprehension: The Role of Math Anxiety, Subjective Numeracy, and Objective Numeracy by Jonathan J. Rolison, Kinga Morsanyi and Ellen Peters in Medical Decision Making
