# Supplementary material for: Understanding Health Risk Comprehension: The Role of Math Anxiety, Subjective Numeracy, and Objective Numeracy
Source: Med Decis Making. 2020 Feb 13;40(2):222–34. doi: 10.1177/0272989X20904725 (PMC7502983; doi:10.1177/0272989X20904725)
Supplement: RiskCompAppendix2019.rjf_online_supp – Supplemental material for Understanding Health Risk Comprehension: The Role of Math Anxiety, Subjective Numeracy, and Objective Numeracy [file RiskCompAppendix2019.rjf_online_supp.pdf]

Appendix A: Materials

Risk comprehension items: Health domain

Imagine that a patient is diagnosed with a cancer known as "Cancer A". Without treatment, there is a 40% chance that they will survive at least one year.

Two treatments are available for people diagnosed with "Cancer A":

Treatment A: The patient's chance of surviving at least one year is increased TO 70%.

Question 1: How many patients among 1,000 who are diagnosed with "Cancer A" will survive at least one year with this treatment? _________ patients

…………………………………………………………………………………………………..

Treatment B: The patient's chance of surviving at least one year is increased BY 25%.

Question 2: How many patients among 1,000 who are diagnosed with "Cancer A" will survive at least one year with this treatment? _________ patients

…………………………………………………………………………………………………..

Question 3: According to estimates of lifetime risk, about 16% (160 out of 1,000 individuals) of people in the general population will develop a cancer known as 'Cancer D', compared with estimates of 48% to 80% (480 – 800 out of 1,000) of people with an alteration in a gene known as ATRW1. In other words, people with an altered ATRW1 gene are 3 to 5 times more likely to develop Cancer D than people without the gene alteration.

Which of the following four options is the most appropriate interpretation of the above statement?

Option 1: Cancer D will develop in 48% to 80% of people who are found to have a ATRW1 gene alteration

Option 2: People who are found to have alterations in the gene called ATRW1 have a 48% to 80% higher chance of developing Cancer D than people who do not have this alteration

Option 3: Cancer D will develop in all people aged 48 to 80

Option 4: People who have an ATRW1 gene alteration will exhibit 48% to 80% of the symptoms associated with Cancer D

…………………………………………………………………………………………………..

Question 4: Two vitamins, A and B, were tested to see if they reduce the risk of neurological disease. For Vitamin A, the experimenters recruited 1,000 volunteers and showed that for 350 of the volunteers, the supplement was effective. For Vitamin B, the experimenters recruited 100 volunteers and showed that for 40 of the volunteers the supplement was effective. Both studies were well powered to test the effectiveness of the vitamins.

Which vitamin do you think was most effective?

Option 1: Vitamin A

Option 2: Vitamin B

Option 3: Vitamin A and Vitamin B are equally effective

…………………………………………………………………………………………………..

Question 5: In a hospital, 10 in every 30 patients who undergo a medical procedure require further treatment and the remaining 20 do not require any further treatment. The last 5 medical procedures carried out in the hospital did not require any further treatment.

What do you think is the most likely outcome for the next patient who undergoes a medical procedure in the hospital?

Option 1: The patient will not require further treatment

Option 2: The patient will require further treatment

Option 3: The patient has equal chances that they will or will not require further treatment

…………………………………………………………………………………………………..

Question 6: According to a recent medical report, 90% of people do not do enough weekly exercise. As a result, 30% of these people are likely to develop a serious health condition in the next 10 years.

According to this medical report, what percentage of people are at increased risk of developing a serious health condition in the next 10 years?

Option 1: 60%

Option 2: 30%

Option 3: 27%

…………………………………………………………………………………………………..

[Participants either completed Questions 7a-12a or Questions 7b-12b]

The chart below describes the attributes of three hospitals (Hospital X, Hospital Y, Hospital Z) in terms of their indicators of cost and quality of care.

Indicators	Hospital X	Hospital Y	Hospital Z
Your out-of-pocket costs	$	$$$	$$
No. of general care beds	550	231	180
Rated quality of hospital food (higher is better)	4.1	1.1	2.0
% of time guidelines for heart attack care are followed	82%	92%	87%
% of time guidelines for pneumonia care are followed	60%	89%	78%
No. of visiting hours per day	11	6	8
No. of registered nurses per 100 patients	18	38	29
Patient references available	Limited	Limited	Limited
Has computer system to prevent medication errors	No	Yes	Limited

Based on the indicators in the chart:

Question 7a: Which hospital is most expensive?

Question 8a: Which hospital is most likely to follow the guidelines for heart attack care?

Question 9a: Which hospital has the fewest registered nurses per 100 patients?

Question 10a: Which hospital is not the most expensive nor does it have the fewest number of registered nurses per 100 patients?

Question 11a: Which hospital does not have a complete computer system to prevent medication errors nor does it follow both guidelines the greatest or smallest % of times?

Question 12a: Which hospital ranks as best based on indicators of the % of times guidelines are followed, the number of nurses per 100 patients, and having a system to prevent medical errors?

The chart below describes the attributes of three hospitals (Hospital X, Hospital Y, Hospital Z) in terms of their indicators of cost and quality of care.

Indicators	Hospital X	Hospital Y	Hospital Z
Your out-of-pocket costs	$	$$$	$$
% of time guidelines for heart attack care are followed	82%	92%	87%
% of time guidelines for pneumonia care are followed	60%	89%	78%
No. of registered nurses per 100 patients	18	38	29
Has computer system to prevent medication errors	No	Yes	Limited

Based on the indicators in the chart:

Question 7b: Which hospital is most expensive?

Question 8b: Which hospital is most likely to follow the guidelines for heart attack care?

Question 9b: Which hospital has the fewest registered nurses per 100 patients?

Question 10b: Which hospital is not the most expensive nor does it have the fewest number of registered nurses per 100 patients?

Question 11b: Which hospital does not have a complete computer system to prevent medication errors nor does it follow both guidelines the greatest or smallest % of times?

Question 12b: Which hospital ranks as best based on indicators of the % of times guidelines are followed, the number of nurses per 100 patients, and having a system to prevent medical errors?

Risk comprehension items: Non-health domain

Question 1: Imagine that a toy shop decides to sell a new product known as “PlayLo”. Without local advertising, there is a 40% chance that the shop will sell enough “PlayLo” in one year to make a profit.

Two local advertising methods are available to toy shops selling “PlayLo”:

Option A: The toy shop’s chance of selling enough “PlayLo” in one year to make a profit is increased TO 70%.

Question 1: How many toy shops among 1,000 that decide to sell “PlayLo” will sell enough in one year to make a profit with this advertising method? __________ toy shops.

………………………………………………………………………………………………..

Option B: The toy shop’s chance of selling enough “PlayLo” in one year to make a profit is increased BY 25%.

Question 2: How many toy shops among 1,000 that decide to sell “PlayLo” will sell enough in one year to make a profit with this advertising method? __________ toy shops.

……………………………………………………………………………………………..

Question 3: According to financial estimates, about 16% (160 out of 1,000 individuals) of people in the general population will not have saved enough for their retirement, compared with estimates of 48% to 80% (480 to 800 out of 1,000) of people who have no retirement saving plan. In other words, people who have no retirement saving plan are 3 to 5 times more likely have insufficient savings than people with a retirement plan.

Which of the following four options is the most appropriate interpretation of the above statement?

Option 1: 48% to 80% of people who have no retirement saving plan will not have saved enough for retirement

Option 2: People who have no retirement saving plan have a 48% to 80% higher chance of not having saved enough for retirement than people who have a retirement saving plan

Option 3: All people aged 48 to 80 will not have saved enough for retirement

Option 4: People who have no retirement saving plan will experience 48% to 80% of the effects associated with not having saved enough for their retirement

……………………………………………………………………………………………..

Question 4: Two vitamins, A and B, were tested to see if they increase milk production in cows. For Vitamin A, the experimenters tested 1,000 cows and showed that for 350 of the cows, the supplement was effective. For Vitamin B, the experimenters tested 100 cows and showed that for 40 of the cows the supplement was effective. Both studies were well powered to test for the effectiveness of the treatments.

Which vitamin do you think was most effective?

Option 1: Vitamin A

Option 2: Vitamin B

Option 3: Vitamin A and Vitamin B are equally effective

……………………………………………………………………………………………..

Question 5: On a stretch of road, 10 in every 30 cars travels over the recommended speed limit and the remaining 20 cars do not travel over the recommended speed limit. The last 5 cars that have passed did not travel over the recommended speed limit.

What do you think is the most likely outcome for the next car that passes along the stretch of road?

Option 1: The car will not travel over the recommended speed limit

Option 2: The car will travel over the recommended speed limit

Option 3: The car has equal chances that it will or will not travel over the recommended speed limit

…………………………………………………………………………………………………..

Question 6: According to a recent financial report, 90% of businesses do not do enough to monitor their expenses. As a result, 30% of these businesses are likely to fail in the next 10 years.

According to this financial report, what percentage of businesses are at increased risk of failing in the next 10 years?

Option 1: 60%

Option 2: 30%

Option 3: 27%

…………………………………………………………………………………………………..

[Participants either completed Questions 7a-12a or Questions 7b-12b]

The chart below describes the attributes of three schools (School X, School Y, School Z) in terms of their indicators of cost and quality of education.

Indicators	School X	School Y	School Z
Your out-of-pocket costs	$	$$$	$$
No. of desks in storage	32	19	8
Rated quality of school food (higher is better)	4.1	1.1	2.0
% of time guidelines for good practice in mathematics education are followed	82%	92%	87%
% of time guidelines for good practice in English literature are followed	60%	89%	78%
No. of teaching hours per day	8	6	7
No. of teaching assistants per 100 students	18	38	29
Student references available	Limited	Limited	Limited
Has computer system to prevent exam assessment errors	No	Yes	Limited

Based on the indicators in the chart:

Question 7a: Which school is most expensive?

Question 8a: Which school is most likely to follow the guidelines for good practice in mathematics education?

Question 9a: Which school has the fewest teaching assistants per 100 students?

Question 10a: Which school is not the most expensive nor does it have the fewest number of teaching assistants per 100 students?

Question 11a: Which school does not have a complete computer system to prevent exam assessment errors nor does it follow both guidelines the greatest or smallest % of times?

Question 12a: Which school ranks as best based on indicators of the % of times guidelines are followed, the number of teaching assistants per 100 students, and having a system to prevent exam assessment errors?

The chart below describes the attributes of three schools (School X, School Y, School Z) in terms of their indicators of cost and quality of education.

Indicators	School X	School Y	School Z
Your out-of-pocket costs	$	$$$	$$
% of time guidelines for good practice in mathematics education are followed	82%	92%	87%
% of time guidelines for good practice in English literature are followed	60%	89%	78%
No. of teaching assistants per 100 students	18	38	29
Has computer system to prevent exam assessment errors	No	Yes	Limited

Based on the indicators in the chart:

Question 7b: Which school is most expensive?

Question 8b: Which school is most likely to follow the guidelines for good practice in mathematics education?

Question 9b: Which school has the fewest teaching assistants per 100 students?

Question 10b: Which school is not the most expensive nor does it have the fewest number of teaching assistants per 100 students?

Question 11b: Which school does not have a complete computer system to prevent exam assessment errors nor does it follow both guidelines the greatest or smallest % of times?

Question 12b: Which school ranks as best based on indicators of the % of times guidelines are followed, the number of teaching assistants per 100 students, and having a system to prevent exam assessment errors?

Objective numeracy scale

[Lipkus et al., 2001]

Question 1: Imagine that we rolled a fair, six-sided die 1,000 times. Out of 1,000 rolls, how many times do you think the die would come up even (2, 4, or 6)?

Rolls out of 1,000 ____________________

Question 2: In the BIG BUCKS LOTTERY, the chance of winning a $10 prize is 1%. What is your best guess about how many people would win a $10 prize if 1,000 people each buy a single ticket to BIG BUCKS?

Persons out of 1,000 ____________________

Question 3: In the ACME PUBLISHING SWEEPSTAKES, the chance of winning a car is 1 in 1,000. What percent of tickets to ACME PUBLISHING SWEEPSTAKES win a car?

% ____________________

Question 4: Which of the following numbers represents the biggest risk of getting a disease?

1 in 100, 1 in 1,000, 1 in 10

Question 5: Which of the following numbers represents the biggest risk of getting a disease?

1%, 10%, 5%

Question 6: If Person A's risk of getting a disease is 1% in ten years, and Person B's risk is double that of A's, what is B's risk?

% ____________________

Question 7: If Person A's chance of getting a disease is 1 in 100 in ten years, and Person B's risk is double that of A's, what is B's risk?

Persons out of 100 ____________________

Question 8: If the chance of getting a disease is 10%, how many people would be expected to get the disease out of 100 people?

Persons out of 100 ____________________

Question 9: If the chance of getting a disease is 10%, how many people would be expected to get the disease out of 1,000 people?

Persons out of 1,000 ____________________

Question 10: If the chance of getting a disease is 20 out of 100, this would be the same as having what chance of getting the disease:

% ____________________

Question 11: The chance of getting a viral infection is .0005. Out of 10,000 people, how many of them are expected to get infected?

Persons out of 10,000 ____________________

[Primi et al., 2016]

Question 12: If three elves can wrap three toys in 1 hour, how many elves are needed to wrap six toys in 2 hours?

Question 13: Jerry received both the 15th highest and the 15th lowest mark in the class. How many students are there in the class?

Question 14: In an athletics team, tall members are three times more likely to win a medal than short members. This year the team has won 60 medals so far. How many of these have been won by short athletes?
